# Supplementary material for: Characterization of the Rat Osteosarcoma Cell Line UMR-106 by Long-Read Technologies Identifies a Large Block of Amplified Genes Associated with Human Disease
Source: Genes (Basel). 2024 Sep 26;15(10):1254. doi: 10.3390/genes15101254 (PMC11507229; doi:10.3390/genes15101254)
Supplement: Supplementary file 1 [file genes-15-01254-s001.zip › genes-3216323-supplementary.pdf]

Supplementary materials:

Gene	Chr	Start	End	OGM UMR Copy No.	ONT UMR:SD DP ratio	UMR TPM	SRR TPM	2-sample T-test *p*-value
**Mdm2**	7	53290660	53315205	4.51	1.09	42	68	NS
**Mdm1**	7	53729603	53766034	4.78	1.16	21	8	NS
**Oxr1**	7	72528750	72965666	3.68	1.34	14	18	NS
**Angpt1**	7	73528345	73783953	4.51	1.48	20	101	0.001
**Csmd3**	7	78747322	80066466	6.5	2.97	<1	<1	NS
**Trps1**	7	81916668	82142733	9.33	3.50	52	22	NS
**Eif3h**	7	83091037	83174451	1.52	5.46	1883	244	**<.001**
**Taf2**	7	86422613	86479616	12.7	4.36	179	20	**<.001**
**Deptor**	7	86514859	86668817	6.57	4.02	11	56	0.002
**Has2 & AS1**	7	88113326	88139337	11.52	3.27	5	71	<.001
**Zhx2**	7	89226358	89374266	15.76	5.23	3	5	NS
**Fam91a1**	7	89969558	90007546	14.79	5.33	306	49	**<.001**
**Tmem65**	7	90336997	90378930	14.09	5.97	117	17	**<.001**
**Rnf139**	7	90439726	90450911	15.68	5.85	212	25	**<.001**
**Myc**	7	93593705	93598633	5.87	7.22	224	64	**<.001**
**Gsdmc**	7	95594015	95606106	7.79	3.40	<1	<1	NS
**Cyrib**	7	95633876	95760588	8.75	3.24	131	25	**<.001**
**Asap1**	7	95786130	96093111	8.96	3.25	175	46	**<.001**
**Adcy8**	7	96417310	96665911	5.55	3.22	5	1	NS
**Efr3a**	7	97552677	97633369	13.62	4.95	199	35	**<.001**
**Kcnq3**	7	97730219	98025652	15.93	5.54	<1	<1	NS
**Phf20l1**	7	98330580	98396526	5.9	3.13	27	22	NS
**Ccn4**	7	98645238	98677253	10.44	2.91	151	27	**<.001**
**Ndrg1**	7	98684487	98725869	15.88	7.47	303	94	**<.001**
**St3gal1**	7	98845270	98913409	25.05	7.62	215	125	NS
**Zfat**	7	99886954	100054288	11.89	4.38	13	3	NS
**Khdrbs3**	7	100837707	100995644	11.72	4.02	316	7	**<.001**
**Col22a1**	7	103730939	103968452	2.53	1.09	17	0	**<.001**
**Trappc9**	7	104521593	104998352	1.77	0.81	11	13	NS
**Chrac1**	7	105013047	105016435	2.72	1.53	37	30	NS
**Mfng**	7	110310810	110328653	3.77	1.29	<1	0	NS

Table S1. Genes from the UMR chromosome 7 amplified region compared by copy number to the control animal using OGM (Column 5), ONT normalized depth ratios of UMR to control (Column 6), RNA sequence TPM for UMR (Column 7), and for SRR osteoblast data from SRA (Column 8). The last column shows the two-sample T-test *p*-value comparing UMR-106 TPM to SRR TPM, with bolded values showing an increased UMR-106 to the control direction of expression relative to SRR. DP: sequence depth; TPM: transcripts per Mb; NS: not significant.
